# Characterising the nicotine metabolite ratio and its association with treatment choice: A cross sectional analysis of Stop Smoking Services in England

**DOI:** 10.1038/s41598-017-17994-8

**Published:** 2017-12-14

**Authors:** Lion Shahab, Emily Mortimer, Linda Bauld, Jennifer A. McGowan, Ann McNeill, Rachel F. Tyndale

**Affiliations:** 1 Department of Behavioural Science and Health, University College London, London, UK; 2 UK Centre for Tobacco and Alcohol Studies, Nottingham, UK; 3 School of Health Sciences, University of Stirling, Scotland Stirling, UK; 4 Addictions Department, Institute of Psychiatry, Psychology & Neuroscience, King’s College London, London, UK; 5 Campbell Family Mental Health Research Institute, Centre for Addiction and Mental Health (CAMH) and Departments of Psychiatry, Pharmacology and Toxicology, University of Toronto, Toronto Ontario, Canada

## Abstract

Pharmacotherapy provision based on Nicotine Metabolite Ratio (NMR) status (slow/normal metabolism) may improve smoking cessation rates. However, it is unclear whether NMR status is consistent across patient characteristics and current treatment choice. Data come from 1,826 participants attending Stop Smoking Services (SSS) across England in 2012/13. Sociodemographic, mental/physical health, smoking and treatment characteristics (nicotine replacement therapy vs. other pharmacotherapy; group vs. one-to-one behavioural support) were assessed. Salivary nicotine metabolites were measured and NMR (3-hydroxycotinine/cotinine) computed, characterising smokers as slow (NMR < 0.31) or normal (NMR ≥ 0.31) metabolisers. Normal metabolisers were older than slow metabolisers (Odds Ratio (OR) = 1.49, 95% Confidence Interval (CI) = 1.32–1.69) but no other characteristics were associated with NMR status. Overall, predictors accounted for only 7.3% of NMR variance. In adjusted analysis, pharmacotherapy type was not associated with NMR status, but normal metabolisers were less likely to use group support (OR = 0.67, 95% CI = 0.51–0.89). NMR status does not vary substantially across sociodemographic characteristics. Given its impact on pharmacotherapy efficacy, the lack of an association with pharmacotherapy choice suggests there is scope to use NMR status to optimise the selection and efficacy of smoking cessation pharmacotherapy. The unexpected association of NMR status with behavioural support should be explored further.

## Introduction

Tobacco use remains a major global health and economic burden, resulting in the premature death of 78,000 smokers in England every year^1^. The principal aim of the National Health Service’s (NHS) ‘Stop Smoking Services’ (SSS) is to provide high-quality, evidence-based smoking cessation interventions to smokers seeking treatment^2^. While approximately 270,000 quit dates are being set each year through these services alone^3^, current CO-validated 4-week cessation rates are 36%^1^, highlighting a need for improvement.

An emerging method for improving cessation outcomes is the personalisation of treatment based on validated biomarkers^4^. One such biomarker is the nicotine metabolite ratio (NMR) – a phenotypic surrogate of nicotine clearance^5^. Nicotine is predominantly metabolised by the liver enzyme CYP2A6^6^ into cotinine, and subsequently exclusively by CYP2A6 into 3′hydroxycotinine^7^. As 3-hydroxycotinine is formation dependent and cotinine has a long half-life, among regular smokers the ratio of 3′hydroxycotinine to cotinine - the NMR - is stable, and independent of time of last cigarette^8^. Due to the prominent role of CYP2A6 in nicotine clearance, the NMR is also a biomarker of total nicotine clearance^5^. The NMR is stable over time^8–10^, and multiple clinical trials have identified differential quit rates for different pharmacotherapies among normal or fast compared with slower metabolisers^11–15^. As NMR incorporates both genetic and environmental influences, it provides a surrogate of nicotine clearance which extends beyond CYP2A6 genotypic measures^16^. It can also be analysed relatively easily from plasma or saliva samples and therefore holds potential clinical utility as a predictive biomarker for cessation outcomes^17^.

The clinical utility of NMR may be further understood through examination of its effectiveness, accessibility, and appropriateness within clinical practice^18^. NMR status (dividing smokers into slow vs. normal metabolisers) has recently been used to prospectively evaluate the efficacy of varenicline versus nicotine patch smoking cessation treatment^15^. Varenicline, compared with NRT, was associated with an increased quit rate among normal but not among slow metabolisers^15^. Lower abstinence rates have also been observed in retrospective analyses of fast or normal metabolisers, compared with slower metabolisers, with the use of NRT patch^11,12,14,19^. In addition, normal compared with slow metabolisers display lower quit rates with behavioural counselling alone^13,20^. Such studies suggest that personalising cessation treatment using the NMR status may be an efficacious and appropriate method of improving the success of attempts to stop smoking.

However, the selection of smoking cessation treatment in naturalistic settings has not yet been explored in relation to NMR status. While NRT aims to reduce withdrawal symptoms by substituting the nicotine source^21^, varenicline both reduces nicotine craving and blocks the rewarding effects of smoking^22^. Given that higher NMR has been associated with greater cigarette craving^19^ and withdrawal during a quit attempt^23,24^, it is possible that smokers are self-selecting the most appropriate cessation aid. That is, over several quit attempts, smokers may learn which pharmacotherapy benefits them most, or stop smoking advisors may already recommend the most appropriate treatment based on characteristics associated with NMR, such as dependence. Further understanding of smokers’ current treatment use would therefore facilitate our understanding of the potential efficacy of the NMR as a biomarker for personalising cessation treatment. For example, as normal metabolisers show greater cessation rates with the use of varenicline, versus nicotine patch^15^, a null association between NMR and treatment choice would suggest that attaining a pre-treatment measure of NMR might improve the selection of – and therefore efficacy of - pharmacological smoking cessation treatment.

In order to further assess the potential suitability of NMR within clinical practice, the reliability of the biomarker with a proposed cut-off to identify slow and normal metabolisers must be considered. While a number of factors, including age^25,26^, ethnicity^27,28^, sex^29^, and nicotine dependence^30^ have been associated with inter-individual variability in NMR, no studies have looked at the association of these factors with NMR status (slow vs normal) and only one clinical trial has explored the association between NMR (but again not NMR status) and multiple sources of variation simultaneously^16^. This is important as any treatment offered will depend on classification into NMR status (e.g. slow vs. normal) and if such characteristics significantly impacted NMR status, this would undermine its clinical utility as an independent marker of treatment success across a range of socio-demographic confounders. As with other clinical markers, an appropriate cut-off point would distribute these characteristics evenly across categories. Moreover, associations with continuous NMR may be statistically significant but not clinically meaningful and in practice vary little by dichotomous NMR status. So far, a range of demographic and environmental factors were found to have little impact on NMR variation, supporting the feasibility of the biomarker as a prospective guide for pharmacological treatment. Replication and extension of these findings in other populations (treatment-seeking smokers) and geographic locations using NMR status is now required. For instance, the association of NMR with mental health status has yet to be explored, despite the increased prevalence of smoking within populations with mental health conditions^31^.

In a large sample of treatment seekers in the UK, the present study therefore aimed to:

1. characterise NMR and NMR status in terms of its association with sociodemographic, smoking, health-related, and treatment characteristics and,
2. explore the relationship between NMR status and treatment choice for smoking cessation, including pharmacotherapy.

## Materials and Methods

### Participants and Procedure

Data from the ‘Evaluating Long-term Outcomes of NHS Stop Smoking Services’ (ELONS) study, a prospective observational examination of 3,045 participants accessing SSS, were used. Participants were recruited between March 2012 and March 2013 from nine regions across England. All participants provided informed consent to take part in this study. Further details regarding the methodology can be found elsewhere^32^. Eligible participants consisted of SSS clients who were not pregnant, were aged 16 or above, and who were willing to provide a saliva sample before cessation treatment began. Additional funding was attained to collect saliva samples upon registration with the SSS^33^. The majority of participants (61.6%, N = 1,875) provided a saliva sample. Those providing a sample were younger, less likely to be female, white British or choose varenicline^32^. They were also more likely to choose group behavioural support. There were no other demographic or treatment differences.

Saliva samples, sociodemographic, smoking, health-related, and treatment characteristics were recorded by NHS SSS staff prior to treatment. Saliva samples were collected on the first clinic visit, prior to setting a quit date, with Sarstedt Salivettes®, posted to University College London and stored in −20 °C freezers. Saliva samples were subsequently shipped to the University of Toronto (N = 1,626) or ABS laboratory (N = 249) for analysis. As an interlaboratory comparison of 100 randomly selected samples assayed at both laboratories showed very high correlation (ρ = 0.91), results from both labs were pooled in this analysis. Ethical approval for the study was provided by the South East Scotland Research Ethics Committee (11/AL/0256) and research complied with the ethical principles on human research, as per the Declaration of Helsinki.

### Measures

#### Sociodemographic measures

Age, sex, ethnicity, and socioeconomic status (SES) were assessed using the ELONS prospective study baseline data collection questionnaire^32^. Due to the limited number of participants from ethnic minority groups, a binary split was used to compare those identifying as White British to all other ethnicities.

The reduced National Statistics Socio-economic Classification (NSSEC) was used as a measure of SES. This conceptualises social class based on occupation/ employment status and has been validated in a number of UK samples^34^, and as a predictor of smoking cessation success^35^. SES was further grouped into higher vs. lower SES, using the NSSEC coding ABC1/C2DE (managerial occupations/manual and unemployed).

#### Smoking Characteristics

Nicotine dependence was measured using the Heaviness of Smoking Index (HSI); a measure of time to first cigarette and cigarettes per day^36^. Participants with a score ≥4 (out of 6), were classified as having high dependence and compared with those with low dependence (<4)^37^. Determination to quit was measured using one item rated with a 4-point Likert scale (ranging from ‘not at all determined’ to ‘extremely determined’) and participants were also asked if they had attempted to quit in the past 12 months (yes/no).

#### Health-related measures

Physical health was assessed using one item; participants selected any applicable medical conditions. Those with at least one medical condition were coded as having poorer physical health, compared with those without. Psychological wellbeing was measured using the WHO-5 wellbeing index^38^. The scale has been credited with reasonable validity, as a method of assessing depression and as an outcome for clinical trials^39^. Participants were scored on a scale of 1–25 (25 representing the highest quality of life), and scores were multiplied by 4 to attain a percentage. A score ≤50% was used to indicate low subjective wellbeing^39^.

#### Stop Smoking Service

Given the likely relationship between treatment choice and SSS location^40^, this was recorded by the relevant practitioners during baseline data collection. For ease of interpretation SSS were divided into North, Midlands and South regions of England.

#### Nicotine Metabolite Ratio

Saliva samples were analysed for cotinine (COT) and trans-3′-hydroxycotinine (3HC) levels using an established LC-MS/MS methodology with a 1 ng/mL limit of quantification (LOQ)^17,41^. The NMR ratio of 3HC/COT was then determined. The ratio has been shown to remain stable over time of day^8^, over a period of months^10^ and while contemplating quitting^9^. Data were examined for analytical shift and reliability, using a reanalysis of samples from each batch (approximately 5% of samples). NMR between replicate analyses was highly correlated (R^2^ = 0.984), there was no association between change in NMR and time between analyses (R^2^ = 0.004) and the percent of difference between analysis replicates varied by less than 10%. Samples with cotinine values below 10 ng/ml were excluded as they likely reflect occasional or light smokers, where the NMR may be unstable. For samples in which COT was above 10 ng/ml, and 3HC was below the limit of quantification, as per convention, the 3HC value was replaced by a dummy value of 0.71 (LOQ/sqrt2) and included in the NMR (i.e. NMR = 0.71/COT)^42^. As plasma and salivary NMR are highly correlated^5^, we used a cut-off for plasma NMR from a previous clinical trial based on population data^15^ to stratify participants into normal (NMR ≥ 0.31) or slow (NMR < 0.3) salivary metabolising status.

#### Treatment characteristics

As this was an observational study, participants were free to choose their treatment after consultation with a SSS practitioner. The elected pharmacotherapy and behavioural support type were recorded by the practitioner. Pharmacotherapy was dichotomised into non-NRT (varenicline or bupropion; these groups were combined as the bupropion sample was too small) vs. NRT product use (single or combined); those receiving no pharmacotherapy and those using NRT with varenicline or bupropion were excluded from analyses of pharmacotherapy. Behavioural support was divided into individual (one-to-one; non-group drop-in) vs. group-based (open/rolling groups; closed groups) support.

### Analyses

Univariate associations between NMR status and sociodemographic, smoking, health-related, and treatment characteristics were assessed using chi-square/t-tests for categorical/continuous variables, respectively. A multi-variable logistic regression was used to explore independent relationships between NMR status (normal vs. slow metabolisers) and each of the sociodemographic, smoking, and health-related characteristics. Separate regression models were used to assess the association between NMR status and pharmacotherapy choice and behavioural support type, controlling for all other covariates. As age did not meet the linearity assumption, it was transformed using the standard deviation of the variable as the scaling factor, and entered into the model as a continuous variable^43^. Similarly, where NMR was treated as a continuous variable, it was log-transformed due to significant non-normality. Sensitivity analyses were conducted using stepwise regression models (backward and forward methods), using NMR as a continuous variable and restricting the NRT sample to NRT patch-only users and non-NRT users to varenicline-only users, given that previous work has focused on these. Main analyses were also repeated defining NMR status by quartiles of individuals (slow metabolisers in first quartile [N = 458] vs. fast metabolisers in forth quartile[N = 452]) and including an NMR status by last year quit attempt interaction term, based on the rationale that individuals may self-select treatment based on experiences with treatment during recent prior quit attempts.

### Data availability

Anonymised datasets generated during and/or analysed during the current study are available from the corresponding author on reasonable request.

## Results

### Participants characteristics and association with NMR and NMR status

**Figure 1:**
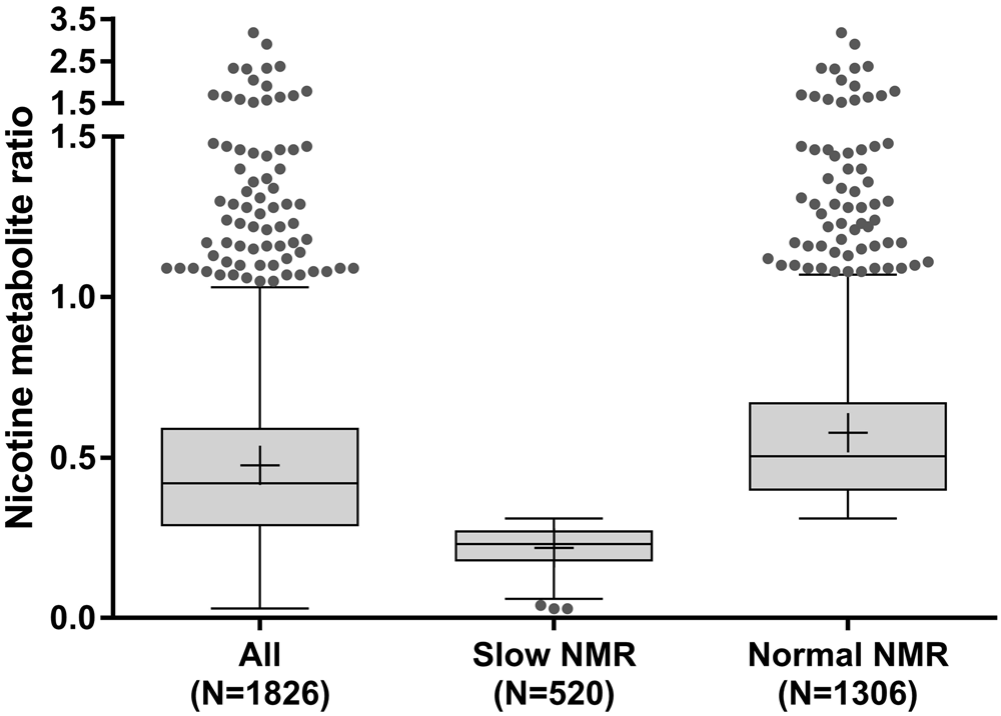
Nicotine metabolite ratio (NMR) by NMR status. Boxplots show median with interquartile range, IQR (25–75%); error bars show Tukey’s whiskers and cross indicates arithmetic mean (geometric means are provided in text); Solid grey circles show outliers.

Of the 1,875 participants for whom saliva samples were obtained, 44 provided samples that contained insufficient saliva or were below the limit of quantification and 5 samples were lost in the post, resulting in 1,826 participants included in the final analysis (Fig. 1). The geometric mean (gM) NMR for all participants was 0.41 (95% Confidence Interval (CI) 0.40–0.42). Of these, 71.5% (N = 1306) were normal (gM = 0.53, 95%CI 0.52 = 0.55) and 28.5% (N = 520) were slow (gM = 0.21, 95%CI 0.20–0.21) metabolisers.

The majority of the sample was white, female and in the C2DE SES group (Table 1). High cigarette dependence was seen in approximately half the sample and most participants reported being extremely or very determined to quit. Just under half had attempted to quit in the previous year and just over half were identified as having poorer psychological wellbeing and/ or poorer physical health (at least one medical condition). Most participants used either NRT or varenicline during their quit attempt and around half sought one-to-one support.

**Table 1:** Baseline characteristics by nicotine metabolite ratio (NMR) status.

	Total (N = 1,826)	Normal NMR (N = 1,306)	Slow NMR (N = 520)	*P*
**Mean (SD) age**	41.7 (14.1)	43.4 (14.0)	37.5(13.6)	<0.001
**% (N) Female**	52.7 (963)	53.8 (703)	50.0 (260)	0.15
**% (N) Higher SES** (ABC1)	23.9 (436)	22.6 (295)	27.1 (141)	0.04
**% (N) White**	95.0 (1,735)	95.8 (1251)	93.1 (484)	0.03
**% (N) Higher dependence score** (HSI ≥ 4)*	48.5 (879)	50.3 (650)	44.1 (229)	0.02
**% (N) Determination to quit†**				0.11
Not at all	8.9 (157)	9.7 (123)	6.7 (34)	
Very determined	42.7 (757)	42.8 (541)	42.6 (216)	
Extremely determined	48.4 (857)	47.5 (600)	50.7 (257)	
**% (N) Last year quit attempt** ^**‡**^	41.2 (730)	41.5 (525)	40.7 (205)	0.79
**% (N) Poor physical health**	55.9 (1,020)	59.1 (772)	47.7 (248)	<0.001
**% (N) Poor wellbeing** ^**§**^	56.8 (992)	58.5 (729)	52.6 (263)	0.03
**% (N) Pharmacological support**				0.53
Single NRT	15.6 (284)	15.6 (204)	15.4 (80)	
Combination NRT	33.7 (615)	33.9 (443)	33.1 (172)	
Varenicline	41.2 (753)	40.6 (530)	42.9 (223)	
Bupropion	1.0 (18)	1.0 (13)	1.0 (5)	
Varenicline and NRT	4.9 (89)	4.7 (61)	5.4 (28)	
Other combination	0.2 (3)	0.2 (3)	0.0 (0)	
None	3.5 (64)	4.0 (52)	2.3 (12)	
**% (N) Group behavioural support**				0.06
Closed group	4.5 (83)	3.8 (49)	6.5 (34)	
Open group	19.4 (354)	18.8 (245)	21.0 (109)	
Drop-in clinic	25.2 (461)	26.1 (341)	23.1 (120)	
One-to-one support	50.5 (922)	51.1 (667)	49.0 (255)	
Other	0.3 (6)	0.3 (4)	0.4 (2)	

HSI = Heaviness of Smoking Index; SES = socioeconomic status; *14 cases missing; ^†^55 cases missing; ^‡^56 cases missing; ^§^80 cases missing

There were some differences between normal and slow metabolisers (Table 1). Normal metabolisers were on average older, more likely to be white, from lower SES groups, with greater cigarette dependence and poorer physical health and psychological wellbeing. However, in adjusted logistic regression analysis, only older age remained a significant predictor of being a normal metaboliser (adj OR 1.49; 95%CI 1.32–1.69; Table 2), with the model accounting for 6.7% of the overall variance in NMR status. In sensitivity analysis using NMR as a continuous variable, the linear regression model explained a similarly small amount of variance (7.3%). Moreover, in addition to older age (B 0.12, 95%CI 0.09–0.15), having a medical condition (B 0.10, 95%CI 0.05–0.16) and being of white ethnicity (B 0.12, 95%CI 0.00–0.24) and female (B = 0.05, 95%CI 0.00–0.11) were also independently associated with a higher NMR (Table 2).

**Table 2:** Associations between nicotine metabolite ratio (NMR) and NMR status and participants characteristics.

	NMR N = 1723*		NMR status (Normal vs. slow metabolisers) N = 1723*	
	B (95%CI)	*P*	Adj. OR (95%CI)	*P*
**Age**	0.12 (0.09–0.15)	<0.001	1.49 (1.32–1.69)	<0.001
**Female** (ref. male)	0.05 (0.00–0.11)	0.05	1.16 (0.93–1.44)	0.19
**Higher SES/ABC1** (ref. C2DE)	−0.04 (−0.10–0.02)	0.18	0.84 (0.66–1.08)	0.17
**White ethnicity** (ref. other ethnicity)	0.12 (0.00–0.24)	0.05	1.47 (0.93–2.34)	0.10
**Higher dependence/HSI ≥ 4** (ref. HSI < 4)	0.03 (−0.02–0.09)	0.20	1.18 (0.94–1.46)	0.15
**Determination to quit**				
Very (ref. not determined)	0.01 (−0.09–0.10)	0.91	0.77 (0.50–1.18)	0.24
Extremely (ref. not determined)	−0.02 (−0.12–0.07)	0.57	0.73 (0.48–1.11)	0.16
**Last year quit attempt** (ref. no attempt)	0.03 (−0.03–0.08)	0.35	1.12 (0.90–1.40)	0.31
**Poor physical health** (ref. good physical health)	0.10 (0.05–0.16)	<0.001	1.22 (0.97–1.53)	0.09
**Poor wellbeing/WHO score** ≤ **50%** (ref. WHO score >50%)	−0.04 (−0.09–0.02)	0.18	1.10 (0.89–1.38)	0.38

Adj. OR = adjusted odds ratio (adjusted for all variables shown); CI = confidence interval; HSI = Heaviness of Smoking Index; SES = socioeconomic status; ref. = reference category; *103 cases missing due to incomplete data.

### Association of NMR status with treatment choice

Results of the regression models exploring adjusted associations of choice of pharmacotherapy type (varenicline/bupropion vs. NRT) and behavioural support type (individual vs. group) with NMR status are presented in Table 3. NMR status was not significantly associated with pharmacotherapy choice. However, relative to slow metabolisers, normal metabolisers were significantly less likely to choose group behavioural support (OR = 0.67, 95%CI = 0.51–0.89). The models explained 9% and 30% of the variance in treatment choice, respectively. Sensitivity analyses using stepwise regression models and using NMR as a continuous variable confirmed these results. No interactions between NMR and SSS on treatment choice were identified, and the lack of association between NMR status or NMR with pharmacotherapy choice was not materially altered when restricting the NRT sample to NRT patch-only users and the non-NRT sample to varenicline-only users. Repeating the main analysis when defining NMR status by quartiles (fast metabolisers [forth quartile] vs slow metabolisers [first quartile]) confirmed results: NMR status was not associated with pharmacotherapy choice (adj OR 0.98; 95%CI 0.88–0.1.06) but relative to slow metabolisers, fast metabolisers were significantly less likely to choose group behavioural support (adj OR 0.81; 95%CI 0.72–0.92). Lastly, there was no significant interaction between NMR status and last year quit attempts on either pharmacotherapy or behavioural support treatment choice.

**Table 3:** Associations between treatment choice and participant characteristics.

	Pharmacotherapy type* N = 1,579‡		**Behavioural support type**† N = 1,717§	
	Adj. OR (95%CI)	*P*	Adj. OR (95%CI)	*P*
**Normal NMR** (ref. slow)	0.96 (0.76–1.21)	0.74	0.67 (0.51–0.89)	0.005
**Age**	0.92 (0.82–1.03)	0.12	0.93 (0.81–1.07)	0.31
**Female** (ref. male)	0.83 (0.67–1.02)	0.07	0.94 (0.73–1.21)	0.64
**Higher SES/ ABC1** (ref. C2DE)	1.53 (1.20–1.95)	0.001	1.10 (0.82–1.47)	0.53
**White ethnicity** (ref. other ethnicity)	5.14 (2.71–9.74)	<0.001	1.19 (0.57–2.49)	0.65
**Higher dependence/HSI ≥ 4** (ref. HSI < 4)	1.10 (0.89–1.36)	0.36	1.27 (0.99–1.64)	0.07
**Determination to quit**				
Very (ref. not determined)	0.89 (0.60–1.30)	0.53	0.63 (0.41–0.98)	0.04
Extremely (ref. not determined)	0.95 (0.65–1.40)	0.74	0.66 (0.43–1.02)	0.06
**Last year quit attempt** (ref. no attempt)	1.02 (0.82–1.25)	0.89	0.93 (0.72–1.20)	0.55
**Poor physical health** (ref. good physical health)	0.69 (0.55–0.86)	0.001	1.14 (0.87–1.48)	0.35
**Poor wellbeing/WHO score** ≤ **50%** (ref. WHO score > 50%)	0.78 (0.63–0.97)	0.02	0.71 (0.55–0.92)	0.009
**SSS Region**				
South (ref. North)	1.50 (1.09–2.07)	0.012	5.52 (3.50–8.71)	<0.001
Midlands (ref. North)	1.73 (1.38–2.16)	<0.001	15.6 (10.8–22.4)	<0.001

^*^Varenicline/bupropion vs. nicotine replacement therapy (reference); ^†^Group support vs. individual support (reference); ^‡^Excludes cases with incomplete data (N = 103) and/or those with either NRT and varenicline/bupropion combination use (N = 86) or no pharmacotherapy use (N = 58); §Excludes cases with incomplete data (N = 103) and/or those with ‘other’ behavioural support (N = 6); Adj. OR = adjusted odds ratio (adjusted for all variables shown); CI = confidence interval; NMR = Nicotine metabolite ratio; HSI = Heaviness of Smoking Index; SES = socioeconomic status; SSS = Stop Smoking Services; ref. = reference category.

## Discussion

This study set out to characterise NMR and NMR status in terms of its association with sociodemographic, smoking, and health-related characteristics and to examine the association between pharmacological/behavioural treatment choice and NMR. Normal vs slow nicotine metabolism was found to be associated with older age, and NMR was also linearly associated with being white, female and having a medical condition but with no other characteristics. Regarding treatment choice, normal NMR status was associated with a reduced likelihood of receiving group behavioural support but not with pharmacological treatment choice.

This study confirms previous research showing a positive association between age and NMR^26^. As it is unlikely NMR increases with age, given that there is no association of age (within adults) with hepatic levels of CYP2A6, the enzyme involved in nicotine and cotinine metabolism, and no impact of age on *in vivo* nicotine or coumarin (a CYP2A6 probe substrate) metabolism^44^, this probably reflects selection bias. That is, it is possible that the greater difficulty in stopping smoking observed in individuals with a normal nicotine metabolism^20,45,46^ results in an increased number of older smokers in this normal NMR subgroup. If this is the case, age would be unlikely to influence the reliability of the biomarker given the suggested direction of the relationship. The fact that age but not poor health remains independently associated with NMR status also suggests self-selection, given that the association of poor health is likely due to the greater number of comorbidities observed in older smokers.

The study also confirms previously observed associations of higher NMR (though not NMR status) with 1) being female, likely due to estrogen effects on inducing CYP2A6, and 2) being of white/Caucasian ethnicity due to a lower frequency of reduced- and loss-of function variants in Caucasians^16,47–49^. It is unclear why continuous NMR (but not categorised NMR status) remains associated with poor physical health, even after controlling for age, and perhaps this reflects some underlying independent genetic association, power issues or artefactual confounding. However, as these characteristics were not related to NMR status and did not differentiate between slow vs. normal metabolisers, explaining less than 8% of the overall variance in NMR, this underlines the clinical utility of NMR status as a consistent and reliable marker across different populations.

This study also set out to explore the relationship between NMR status and treatment choice. No association between NMR status and the use of either non-NRT or NRT-based pharmacotherapy was detected (including NRT patch alone), implying that there is no treatment self-selection as a function of NMR status. This is further confirmed by the fact that experience of recent quit attempts did not affect the association (or lack thereof) of NMR status with treatment choice. Given the improved effectiveness of non-NRT over NRT-based pharmacotherapy in normal but not slow metabolisers^15^, this suggests there is room to improve treatment outcomes by personalising treatment. Specifically, as the numbers needed to treat reduces from 26 to 5 for NRT to non-NRT pharmacotherapy in normal metabolisers, this group could be treated preferentially with varenicline, bupropion or similar medication, something which is currently not the case. Good quality clinics may want to explore their capacity to test for this biomarker when assessing clients as a way of informing pharmacotherapy choice

Interestingly, normal metabolisers were less likely to choose group behavioural support. As normal metabolizers were on average older, we analysed the data both as a confounder (Table 3) and as an age by NMR status interaction term (not significant), suggesting age was not the explanation. Slower compared with faster metabolisers have been reported to benefit more from behavioural counselling alone^13,20^. While the cause of this relationship is largely unknown, normal or fast nicotine metabolism has been linked with longer smoking duration^46^, increased cigarette craving during a quit attempt^19,20,50^, and greater nicotine dependence^37^. Given the greater difficulty in stopping smoking in this group, it could therefore be speculated that normal metabolisers may perceive an additional benefit from individual support compared with group support, which allows for flexible consultations that can be tailored to the clients personal situation and their experience of previous quit attempts^51^. In addition, the increased need for pharmacotherapy in this group may underlie this association as pharmacotherapy is more likely to be accessed via community practitioners (e.g. General Practitioners, pharmacists) who predominantly offer individual support. By contrast, specialist stop smoking clinics provide a range of behavioural support including group counselling which may not always involve pharmacotherapy^32^. Overall, however, the study that provided our data found that group support was more effective in supporting smokers to quit^32^, which may suggest that normal metabolisers should be actively encouraged to consider group support even if their natural preference may be for one-to-one options.

This study has a number of limitations. The cross-sectional design does not allow us to make causal or directional claims. Moreover, while we did include a variety of characteristics to investigate the association with NMR, a number of putative factors were not measured and some variables were only assessed with a single item. Although treatment site was controlled for in analysis, the extent to which the available resources and individual treatment providers within the region influenced treatment choice could not be determined directly. Yet, treatment site is unlikely to have influenced NMR and therefore this analysis. Lastly, while the initial sample collected was largely representative of smokers seeking treatment in the UK, there were some marked demographic and treatment differences between those who provided a sample and those who did not.

## Conclusions

Although significant associations between NMR and ethnicity, physical health and gender were identified, these sociodemographic, smoking, and health-related characteristics did not greatly influence variability in NMR and did not confound allocation to either slow or normal NMR status. This suggests that NMR status is relatively unrelated to these variables, functioning consistently across different populations, thus increasing its potential for use in clinical practice. The unexpected association of NMR status with behavioural support should be explored further in clinical trials and studies of real-world data. Given the association of NMR with pharmacotherapy efficacy, the finding that NMR status is currently not associated with pharmacotherapy choice suggests there is a need for providers to tailor smoking cessation therapies based on rate of nicotine metabolism as patients are not naturally selecting the option that has the highest potential efficacy for them.

## Electronic supplementary material

Strobe Checklist
